# Basal Insulin Inadequacy versus Failure – Using Appropriate Terminology

**DOI:** 10.17925/EE.2015.11.02.79

**Published:** 2015-08-19

**Authors:** Sanjay Kalra, Yashdeep Gupta

**Affiliations:** 1. Department of Endocrinology, Bharti Hospital, Karnal, India; 2. Department of Endocrinology, All India Institute of Medical Sciences, New Delhi, India

**Keywords:** Basal insulin failure, basal insulin inadequacy, degludec, detemir, glargine, neutral protamine Hagedorn, type 2 diabetes, terminology

## Abstract

This editorial focuses on appropriate terminology related to basal insulin therapy. The authors analyse current usage of ‘basal insulin failure’, and propose ‘basal insulin inadequacy’ as a better descriptor for persons not responding to basal insulin alone. The pharmacokinetic and pharmacodynamic differences between various basal insulin preparations are highlighted. Based upon these, a drug-specific definition for insulin inadequacy is suggested, instead of a generic class-based labelling.

Basal insulin therapy is recommended as a first-line injectable therapy in persons with type 2 diabetes who do not respond to metformin monotherapy. Insulin works to control fasting glycaemia, and it is expected that metformin (with or without other drugs) will suffice apropos post-prandial euglycaemia. In many patients, however, basal insulin is unable to achieve adequate glycaemic control. This has been termed basal insulin failure. However, in the light of newer developments, we suggest a more appropriate term, basal insulin inadequacy, and discuss how it can be used.

## Basal Insulin Failure

According to the current American Diabetes Association (ADA)/ European Association for Study of Diabetes (EASD) guidelines, change of basal insulin therapy is indicated if the treatment strategy fails to achieve normal glycated haemoglobin (HbA_1c_) in spite of adequate fasting control, or if >0.5 μ/kg/day of basal insulin is required.^1^ Basal insulin failure has earlier been defined as the inability to achieve a pre-decided target glycaemic control, after optimisation of lifestyle modification measures and maximal titration of basal dose beyond which unacceptable hypoglycaemia will occur.^2^

## Basal Insulin Inadequacy

The current nomenclature implies that basal insulin has ‘failed’, while actually it may have succeeded in achieving fasting euglycaemia. Thus a more appropriate terminology is ‘basal insulin inadequacy’. This conveys a more accurate message that basal insulin is inadequate for the particular patient’s need. Inadequacy avoids the negative connotation associated with the word ‘failure’. It does not pass judgmental opinion on either the patient’s efforts to manage lifestyle or the physician’s choice of therapeutic strategy.

## Basal Insulin Dissimilarity

All basal insulins are not alike. Each basal insulin and basal analogue has a unique structure, which contribute to specific pharmacokinetic and pharmacodynamic characteristics.^3^ These properties allow a systematic listing of basal insulin as intermediate-, long- and ultra-long-acting molecules (see *Tables 1* and *2*). The differences in duration of action, glycaemic variability and risk of hypoglycaemia, specifically nocturnal hypoglycaemia, may allow for substitution of one basal insulin for another, in case adequate control is not achieved with a particular preparation. Thus, a new strategy for intensification of therapy is available for persons not responding to basal therapy: a switch to a longer-acting basal insulin.

**Table 1:** Classification of Basal Insulins and Insulin Regimes

Intermediate acting	Neutral protamine Hagedorn
Long acting	Glargine, detemir
Ultra-long acting	Degludec
Of historical interest	Lente, semi-lente, ultra-lente

Such a therapy is supported by mechanistic studies, randomised controlled trials (RCTs) and meta-analysis. Insulin degludec, for example, has been shown to have a longer half-life and duration of action, with significantly less glycaemic variability than glargine.^4^

RCTs and meta-analyses report a significantly lower incidence of hypoglycaemia and nocturnal hypoglycaemia, while achieving better fasting glucose control, in persons randomised to insulin degludec compared with glargine.^5–8^ Refractory patients, switched from glargine to degludec, have also been reported to achieve good glycaemic control in clinical practice.^9,10^ Cost-effectiveness of such a shift is also found to be beneficial.^11,12^ Thus, the clinical phenomenon of ‘basal insulin inadequacy’ may be drug-specific. Inability of a particular basal insulin to achieve adequate glycaemic control does not imply that all basal insulins will be inadequate for the purpose. Applied to a patient not responding to glargine, the phrase ‘basal insulin inadequacy’ may not be valid for all basal insulins.

**Table 2:** Comparison of Basal Insulins and Insulin Analogues

Insulin	NPH	Glargine	Detemir	Degludec
Structure	Addition of protamine to unmodified human insulin, in quantity sufficient to complex all insulin molecules	Acidic long-acting analogue with substitution of asparagine with glycine at A21, and addition of two arginine residues at B30	Deletion of threonine at B30 and addition of aliphatic fatty acid to lysine at B29	Deletion of threonine at B30 and addition of 16-chain carbon fatty di-acid to lysine at B29 with glutamic acid as a spacer
Number of amino acids	51	53	50	50
Appearance	Cloudy	Clear	Clear	Clear
Onset of action	2–3 h	1–4 h	1–4 h	–
Half-life	–	12.5 h	12.5 h	25 h
Duration of action	Up to 18 h	Up to 24 h	16-24 h	Up to 42 h
Mechanism of protraction	Slow dissociation	Precipitates form in subcutaneous tissue and dissociate slowly	Reversible binding with albumin and slow dissociation of multimers	Multi-hexamers form in subcutaneous tissue and dissociate slowly
Binding affinity to IGF-I receptor (human insulin 100)	100	641±51	18±2	2
Binding affinity to insulin receptor (human insulin 100)	100	86±3	16±1	13-15
Variability	High	Medium	Low	Very low
Ratio of exposure over first 12 h and second 12 h after injection	–	60:40	50:50	50:50
Risk of hypoglycaemia	May occur	Low	Low	Minimal
Risk of nocturnal hypoglycaemia	May occur	May occur	Minimal	Minimal
Risk of weight gain	Yes	Yes	No	Minimal
Injection-site reactions	Rare	Possible, because of acidic pH	Rare	Rare
Miscibility with rapid-acting insulin	Yes	No	No	Yes
Miscibility with GLP1RA	No	Yes (with lixisenatide)	No	Yes (with liraglutide)
Frequency of administration	Once to twice daily	Once daily	Once to twice daily	Once daily
Timing of administration	At same time every day	At same time every day	At same time every day	At any time of the day

GLP1RA = glucagon-like peptide-1 receptors agonists; IGF-1 = insulin-like growth factor 1; NPH = neutral protamine Hagedorn. Modified with permission from Kalra S, Newer basal insulin analogues: degludec, detemir, glargine, *J Pak Med Assoc*, 2013;63(11):1442-4.^3^

We therefore suggest the following terminology and definitions:

- Basal insulin inadequacy may be defined as the inability of all basal insulin preparations, prescribed alone or in combination with various oral glucose-lowering drugs, to achieve pre-decided glycaemic targets, without causing unacceptable hypoglycaemia or weight gain, in spite of optimal lifestyle modification and maximal dose titration.
- Intermediate-, long-acting and ultra-long-acting insulin inadequacy may be used to describe persons who do not respond to maximal doses of NPH, glargine and detemir and degludec, respectively.
